# Effectiveness of the blended-care lifestyle intervention ‘PerfectFit’: a cluster randomised trial in employees at risk for cardiovascular diseases

**DOI:** 10.1186/s12889-018-5633-0

**Published:** 2018-06-19

**Authors:** Tessa A. Kouwenhoven-Pasmooij, Suzan J. W. Robroek, Roderik A. Kraaijenhagen, Pieter H. Helmhout, Daan Nieboer, Alex Burdorf, M. G. Myriam Hunink

**Affiliations:** 1 Department of Epidemiology, Erasmus MC, University Medical Center Rotterdam, Na2818, Postbus 2040, 3000 CA Rotterdam, The Netherlands; 2 Department of Public Health, Erasmus MC, University Medical Center Rotterdam, Rotterdam, The Netherlands; 3 Department of Occupational Health, Erasmus MC, University Medical Center Rotterdam, Rotterdam, The Netherlands; 4 NDDO Institute for Prevention and E-health Development (NIPED), Amsterdam, The Netherlands; 5 Staff Joint Health Care Division, Command Service Center, Ministry of Defense, Utrecht, The Netherlands; 6 Department of Radiology, Erasmus MC, University Medical Center Rotterdam, Rotterdam, The Netherlands; 7 Center for Health Decision Sciences, Harvard T.H. Chan School of Public Health, Harvard University, Boston, USA

**Keywords:** Life style, Risk assessment, Cardiovascular diseases, Body weight, eHealth, Motivational interviewing

## Abstract

**Background:**

Web-based lifestyle interventions at the workplace have the potential to promote health and work productivity. However, the sustainability of effects is often small, which could be enhanced by adding face-to-face contacts, so-called ‘blended care’. Therefore, this study evaluates the effects of a blended workplace health promotion intervention on health and work outcomes among employees with increased cardiovascular risk.

**Methods:**

In this multicentre cluster-randomised controlled trial (PerfectFit), 491 workers in 18 work units from military, police, and a hospital with increased cardiovascular risk were randomised into two intervention groups. The limited intervention (*n* = 213; 9 clusters) consisted of a web-based Health Risk Assessment with advice. In the extensive intervention (*n* = 271; 8 clusters), coaching sessions by occupational health physicians using motivational interviewing were added. One cluster dropped out after randomisation but before any inclusion of subjects. Primary outcome was self-rated health. Secondary outcomes were body weight, body mass index (BMI), work productivity, and health behaviours. Follow-up measurements were collected at 6 and 12 months. Effect sizes were determined in mixed effects models.

**Results:**

At 12 months, the extensive intervention was not statistically different from the limited intervention for self-rated health (4.3%; 95%CI -5.3-12.8), BMI (− 0.81; 95%CI -1.87-0.26) and body weight (− 2.16; 95%CI -5.49-1.17). The within-group analysis showed that in the extensive intervention group body weight (− 3.1 kg; 95% CI -2.0 to − 4.3) was statistically significantly reduced, whereas body weight remained stable in the limited intervention group (+ 0.2 kg; 95% CI -1.4 to 1.8). In both randomised groups productivity loss and physical activity increased and excessive alcohol use decreased significantly at 12 months.

**Conclusions:**

There were no effects on self-rated health, body weight, and BMI. However, within the group with web-based tailored Health Risk Assessment including personalized advice body weight reduced significantly. Adding motivational coaching is promising to reduce body weight.

**Trial registration:**

Retrospectively registered at the Netherlands Trial Registry with number NTR4894, at Nov 14 2014.

**Electronic supplementary material:**

The online version of this article (10.1186/s12889-018-5633-0) contains supplementary material, which is available to authorized users.

## Background

Non-communicable diseases are a major burden all over the world. Health risk behaviours, such as smoking, unhealthy diet, and physical inactivity, are associated with obesity and cardiovascular diseases (CVD) [1], and are also responsible for substantial health care costs and indirect costs in the workplace [2, 3]. In ageing societies, health promotion programmes that contribute to healthy ageing of the workforce are increasingly important [4, 5].

In western countries individuals are required to work longer due to increasing retirement ages. There is a societal need to work longer in good health. The workplace has been identified as a promising setting for health promotion, because of the possibility to reach large groups and the presence of a natural social network. Two approaches that have shown promise in improving unhealthy behaviours are a web-based Health Risk Assessment (HRA) and individual counselling by using Motivational Interviewing (MI). Some studies have demonstrated that a web-based HRA stimulated individuals to undertake health-promoting activities and achieved a healthier lifestyle, a decreased CVD risk, and reduced absenteeism in both work [6–8] and primary care settings [9]. Motivational interviewing as a coaching technique has shown beneficial effects on behavioral and biomedical outcomes in individuals with increased CVD risk [10–12], with maintenance of the effect at 12 months follow-up [13]. Web-based HRAs are appealing as they could reach large populations without extensive human interaction [9]. The major shortcoming of a purely web-based approach is low sustained participation [14]. A systematic review reported that maintenance of behavioral changes was higher in interventions with face-to-face contact than those without [15]. Addley et al. [16] recently suggested adding face-to-face contact in a health mentoring programme to an HRA to achieve enhanced benefits on health and work outcomes. Motivational interviewing is recommended as face-to-face communication strategy by The American Heart Association [17]. By nonjudgementally addressing a person’s innate needs and values during different phases of change, MI-approaches contribute to sustainable change [18]. MI has potential benefits above tailored advice, but it requires intensive human and financial resources [19, 20]. Although optimizing personalized prevention by blending a web-based HRA and face-to-face motivational counselling seems promising, little is known about whether adding these components to workplace health promotion programmes will increase their effectiveness.

The hypothesis for this study was that a web-based HRA combined with MI improves the motivation for behaviour change, and subsequently improves health behaviour and health more than a web-based HRA without MI. The main aim of the current study was to evaluate the effects of adding MI-coaching to a web-based HRA including tailored advice on health and work outcomes among employees with increased cardiovascular risk in the military workforce, the police organisation and an academic hospital.

## Methods

### Study design

The PerfectFit study was designed as a cluster-randomised controlled trial with randomisation carried out at organisational units within three large organisations with a workforce with physically and mentally demanding jobs: the military (9 clusters), the police force (3 clusters), and an academic hospital (5 clusters) [21]. The cluster design ensured that occupational health physicians (OP) delivering the intervention were only active within a single study arm. Reporting of the study was performed according to the CONSORT extension for cluster trials [22] (Additional file 1: Table S1 and S2).

Baseline measures were obtained from all participants between 2012 and 2014 after written informed consent was given. Web-based follow-up questionnaires were collected at 6 and 12 months. Anthropometric and blood measurements were repeated at 12 months. An extensive description of the study design and baseline characteristics is provided elsewhere [21]. The Medical Ethics Committee of Erasmus MC Rotterdam (METC) approved the study with number MEC-2012-459. The study was registered in the Netherlands Trial Registry with number NTR4894.

The academic hospital was included in the trial after trial commencement, for which approval of the METC was obtained. Reasons were the loss of one military cluster, leading to less inclusions than expected.

### Randomisation, blinding, and sample size calculation

To guarantee allocation concealment, randomisation was performed by a researcher who was not otherwise involved in the trial, using R version 3.0.1.

A total of 18 clusters were randomised, and since 1 cluster dropped out prior to any inclusion of employees, analyses were performed on 17 clusters. Participants were included by 21 OPs. For the hospital, the first MI-session was performed by the OP and follow-up sessions by a lifestyle coach. Due to our design, OPs, lifestyle coaches and participants were not blinded.

The sample size calculation took into account the cluster design [23] with an estimated intracluster correlation coefficient of 0.05. We estimated that approximately 220 participants per study arm were needed to demonstrate an effect size of 10% in self-rated health between the two groups [21].

### Participants

A total of 652 employees of 40 years and over attended the ‘cardioscreening’ at occupational health centers, which consisted of a short web-based questionnaire, anthropometric measurements, and blood measurements, and is described in detail elsewhere [21]. Inclusion criteria were: 1) having angina or myocardial infarction in first degree relatives; 2) not meeting the Dutch physical activity norm of exercising five times a week at moderate intensity for at least half an hour; 3) smoking; 4) self-reported diabetes mellitus or random glucose ≥ 11.1 mmol/l; 5) obesity (BMI ≥ 30 kg/m^2^ and / or waist circumference ≥ 102 cm for men or BMI ≥ 30 kg/m^2^ and/or ≥ 88 cm for women); 6) hypertension (diastolic value > 90 mmHg or a systolic value > 140 mmHg) or the use of antihypertensive drugs); and 7) dyslipidaemia (total cholesterol ≥ 5 mmol/l or LDL cholesterol ≥ 2.5 mmol/l or triglycerides: ≥ 1.7, mmol/l or HDL cholesterol: ≤ 1.0 mmol/l). Elevated risk for CVD was defined as having at least one of the inclusion criteria. Of the 652 screened individuals, 91.7% (*n* = 598), had an elevated risk for CVD and were invited by the OP to participate in the study of which 491 (82.1%] subjects provided informed consent.

### Interventions

The limited (control) intervention programme consisted of the following elements:

- A web-based HRA, including tailored and personalized feedback based on the participant’s risk profile, with suggestions for particular health promotion activities, available within each organisation.
- An electronic newsletter, providing information on the intervention (PerfectFit) and general information on a healthy lifestyle, which was sent to email-addresses using newsletter-software [24]*,* every 2 to 3 months during the study period.

In the extensive intervention group, the intervention was extended with:

c) Seven individual coaching sessions (3 face-to-face and 4 by telephone) with an OP, together with more personalized suggestions for health promotion activities based on motivational elements in the HRA, and an additional motivational paragraph in the newsletters.

During the coaching sessions, the OP applied a client-centered counselling style with MI techniques such as asking open questions, reflecting, supporting, and raising ambivalence. Starting point of the counselling was problem feedback [25] by discussing the person’s CVD risk profile and motivation to change health behaviour, which was integrated with important life goals and values. All OPs in the extensive intervention group received a basic training in MI of 3 full days and 3 follow-up coaching sessions of 4 h.

### Outcome measures

The primary outcome measure was self-rated health, assessed by the first question of the Short Form 36 Health Survey (SF-36) [26] (‘Overall, how would you rate your health?’) with 5 answers, ranging from ‘very poor’ to ‘very good’. Answers were dichotomized in ‘less than good’ and ‘good or very good’ health, as was done in the power calculation [21].

The secondary outcome measures were body weight, BMI, work performance, and health behaviours. Body weight was expressed in kilograms and Body Mass Index (BMI) in kg/m^2^. ‘Obesity’ was defined as BMI ≥30 kg/m^2^. Body height and weight were measured at each OP’s clinic at baseline and at 12 months with calibrated scales available at their occupational health clinics.

Work performance was estimated by work ability, sickness absence, and productivity loss at work. Work ability was measured with the first question of the Work Ability Index (WAI) questionnaire [27], rating a worker’s current work ability relative to the best work ability during life on an 11-point scale ranging from 0 (unable to work) to 10 (current work ability equals best work ability ever). Sickness absence in days off work due to illness was determined by the 5th question of the WAI, and answers were categorized into no sickness absence (0 days), short-term (1–9 days), and long-term (≥10 days). Productivity loss at work was assessed with the short version of the Work Limitations Questionnaire (WLQ-8) [28–30], consisting of four dimensions: physical (2 items), time management (2 items), mental-interpersonal (2 items), and output demands (2 item). Individuals rated impairments on a 5-point scale from ‘always’ to ‘never’, or ‘does not apply to my job’. The WLQ-8 coding algorithm produced a summary score representing the percentage of productivity lost at work over the last 2 weeks due to health reasons.

Health behaviours addressed were physical activity (PA), fruit and vegetables, smoking, alcohol, and perceived stress. Compliance with the Dutch guideline on physical activity (PA) [31] was measured by asking ‘are you at least 5 days a week, for at least 30 minutes per day, physically active at a moderate intensity (i.e. with a slightly increased heart rate and breathing rate, such as in vigorous walking or cycling)?’ (yes/no). Compliance with the guidelines on a healthy diet [32] was assessed by self-reported daily intake of vegetables on a 6-point scale (‘no vegetables’ to ‘4 or more spoons of 50 grams each per day’), and fruits on a 7-point scale (‘never’ to ‘twice a day’). The recommendation was not met if less than 200 g of vegetables and less than 2 pieces of fruit were consumed every day. Smoking was measured with the question ‘do you smoke?’ (yes/no). Alcohol intake was measured by asking the number of alcohol-units consumed per week with a 7-point scale (1 = ‘less than 1 glass per week’, 7= ‘43 to 50 glasses per week’). The guideline was not met if more than 7 (women) or 14 (men) glasses per week were consumed [32]. The level of stress was measured by the INTERHEART-questionnaire [33]. We defined ‘high stress level’ as several periods or permanent stress at work or at home, severe financial stress, or 2 or more life events in the past year [33]. With the exception of healthy diet and high stress level, health behaviours were measured at 6 and 12 months by a short web-based questionnaire.

### Delivery of the intervention

The quantity of the intervention delivered was expressed by number of face-to-face and telephone MI sessions, and the mean duration in minutes of MI counselling received. The fidelity of the intervention, i.e. the quality of MI, was determined by audio-records of a session every 3 months per OP [34]. Recorded sessions were transcribed verbatim and analysed using the validated Motivational Interviewing Treatment Integrity code (MITI) version 3.1.1 [35]. Coding was done by two experienced MI-coaches (TK, MW) who were also familiar with the scoring technique. Quality of MI was expressed by the MITI global score ‘empathy’ and the behaviour-count ‘MI-adherence’, since these may be predictive of successful client outcome [34]. ‘Empathy’ referred to the OP’s efforts in understanding the client’s perspective, ranging from 1 (low) to 5 (high). ‘MI-adherence’ referred to provision of information (teaching or feedback on personal information) in a MI-consistent way, and was calculated as percentage of MI-adherent remarks. MI quality was based on 35 recordings, ranging from 1 to 4 recordings per OP.

### Data analyses

Differences between the limited and extensive intervention groups at baseline were evaluated with Chi-Square tests for dichotomous variables and ANOVA-tests for continuous variables. No adjustments for clustering were done because the intracluster correlation was low.

All analyses were performed according to the intention-to-treat principle, including all participants regardless of whether or not they received the intervention according to protocol. Non-response analyses were conducted to determine whether drop-out was associated with any baseline characteristics or with the type of intervention. Non-response was defined as no response to the questionnaire at 6 or 12 months. The changes in health, work outcomes and health behaviours within each group were evaluated at 6 and 12 months using paired T-tests for continuous variables and McNemar’s test for categorical variables. No adjustments were done because the intracluster correlation was low.

A random intercept for organisation cluster was used to take into account the clustered design. The intercepts are allowed to vary between clusters. Furthermore, the intervention effect was adjusted for baseline health, work outcomes, and health behaviours, sex, age, and education, which were added as fixed effects. Missing values of adjustment variables were imputed using chained equations using the mice package in R. Since the percentage of missing values was low we used single imputation. The intra-cluster correlation was assessed to evaluate the within cluster variation, and was 0.08 at the highest, implying that the clustering had little effect on the results. Data were analysed using SPSS Statistics version 21. Imputation of missing baseline characteristics we used the mice package in R. Mixed effects models were fitted using the lme4 package.

## Results

**Fig. 1:**
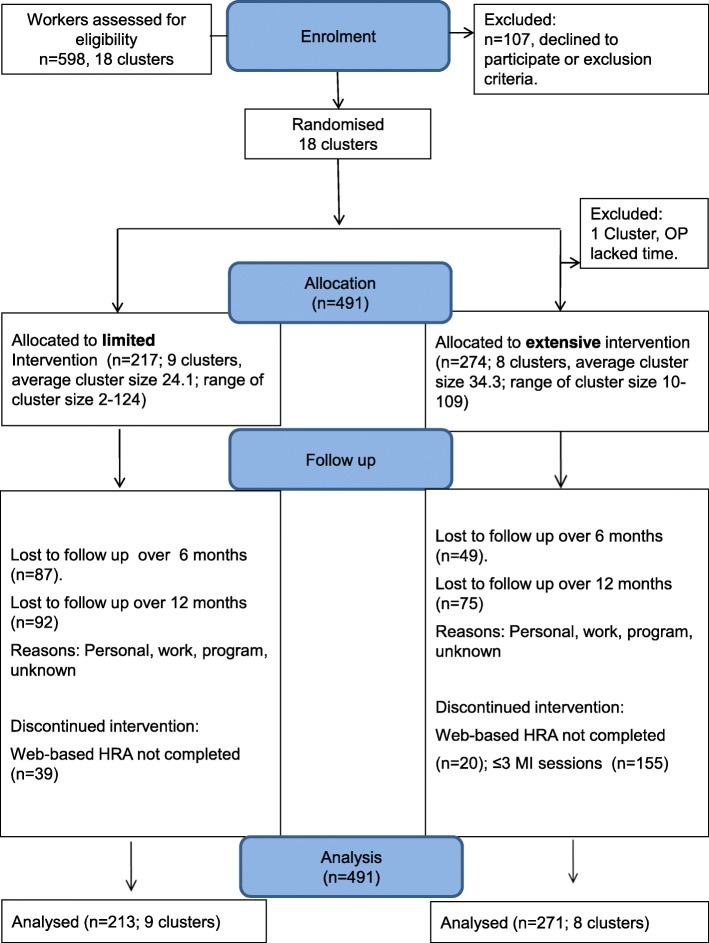
CONSORT flow chart of clusters and participants within the trial

In Fig. 1 the flow of participants is shown with 9 clusters (*n* = 217) in the limited and 8 clusters (*n* = 274) in the extensive intervention group.

Our study population was 50.8 years on average, predominantly male with intermediate or high education (Table 1). Overall, 18.3% considered themselves to be in less than good health, and health behaviours showed that 65.4% did not meet the Dutch physical activity guidelines and 86.6% did not meet the healthy diet guidelines. In total, 16.9% of the participants smoked, 11.8% used excessive alcohol, and 37.4% reported a high stress level. Since both randomised groups differed in age, sex, and educational level, all statistical analyses were fully adjusted for these factors. No adverse events of the intervention were reported.

**Table 1:** Baseline characteristics of the study population (*n* = 491)

Characteristics	Limited intervention *n* = 217 (44.2%)	Extensive intervention *n* = 274 (55.8%)	*P* value	Missings *n* (%)
Individual characteristics:				
Age, years (mean, SD)	51.62 (6.0)	50.19 (5.6)	0.003	1 (0.2)
Male (*n*,%):	166 (76.5)	233 (85.0)	0.016	0
Level of education (*n*,%):			0.009	20 (4.1)
Low	34 (17.9)	33 (12.6)		
Medium	116 (61.1)	140 (53.6)		
High	40 (21.1)	88 (33.7)		
Health characteristics:				
Self-rated general health:				
Less than good	32 (17.2)	58 (22.3)	0.185	45 (9.2)
BMI (kg/m2) (mean, SD)	26.9 (3.4	27.5 (3.6)	0.066	15 (3.1)
Work characteristics:				
Work ability (mean, SD)	7.91 (1.61)	7.92 (1.47)	0.946	50 (10.2)
Sickness absence (*n*,%)			0.894	53 (10.8)
0 days	92 (50.3)	123 (48.2)		
1–9 days	72 (39.3)	106 (41.6)		
≥ 10 days	19 (10.4)	26 (10.2)		
Productivity loss at work (%, SD)	2.93 (3.46)	2.97 (3.62)	0.916	103 (21.0)
Health risk behaviour^a^, *n* (%):				
Lack of physical activity	133 (72.7)	188 (72.3)	0.932	48 (9.8)
Unhealthy diet^b^	173 (94.5)	252 (97.7)	0.082	50 (10.2)
Smoking	28 (12.9)	55 (20.1)	0.071	0
Excessive alcohol use^c^	26 (15.9)	32 (14.0)	0.617	99 (20.2)
High stress level^d^	59 (32.2)	106 (41.1)	0.059	50 (10.2)

*BMI* body mass index, *SD* standard deviation

^a^Defined as non-adherence to Dutch guidelines at baseline

^b^Unhealthy diet is defined as eating less than 200 g vegetables per day, and eating less than 2 pieces of fruit per day

^c^Meeting the alcohol guideline, which is not drinking more than 1 (women) or 2 (men) glasses of alcohol a day

^d^High stress level is defined as several periods or permanent stress at work or at home or severe financial stress or 2 or more life events (Ref. Lancet 2004 Rosengren)

The response was 72% at 6 months and 66% at 12 months. The extensive intervention approach contributed to retaining individuals in the study, and more MI sessions improved adherence (Additional file 2: Table S3). Response was higher among those with a higher workability (8 versus 7.5) and less productivity loss at work. There were no differences between OPs in both intervention groups in years affiliated with the organization (mean 11.9, range 0.5 to 45.0) and working hours per week (mean 36.7, range 32.0 to 40.0). None of the OPs in the limited group had ever been MI-trained.

Table 2 shows the changes in health, work performance, and health behaviours during the study, and the effectiveness of the intervention. The proportion of subjects in ‘less than good health’ remained stable over time in the extensive group, but increased slightly in the limited group. After 12 months a statistically significant difference of 0.81 BMI points and 2.16 kg body weight was observed, favouring the extensive intervention. After adjustment for important individual characteristics the difference in BMI and body weight became insignificant, illustrating the imbalance in study populations in both programmes. Hence, a within-effect analyses was also conducted, showing that in the extensive intervention group there was a significant reduction of 0.69 kg/m^2^ (3.1 kg body weight) in the extensive group, whereas no changes were found in the limited group. There were no significant differences in health behaviours between the groups. The proportion of individuals meeting the physical activity guidelines increased by more than 50% in both randomised groups. Alcohol consumption was equally reduced by 10% after 12 months in both randomised groups. In both randomised groups the prevalence of smoking reduced somewhat. A reduction of 1.6% points in long-term sickness absence after 6 months and 7.2% points after 12 months was found in the extensive group, although not statistically significant.

**Table 2:** Changes in health, work and health risk behaviour after 6 and 12-months follow-up in the limited and extensive intervention groups. The estimated effect is the difference between the extensive intervention vs. the limited intervention, adjusted for baseline characteristics. For example, the negative difference for BMI implies that the extensive intervention had a greater effect in reducing BMI

	Effect follow-up minus baseline^a^				Estimated effect^b^ (difference) between intervention groups (95% CI)	
	Limited intervention		Extensive intervention			
Outcome:	6 months	12 months	6 months	12 months	6 months	12 months
Health characteristics:						
General health (%) less than good	3.1	4.1	-1.8	0	2.6 (−8.4;9.2)	4.3 (− 5.3;12.8)
BMI (kg/m^b^), (mean, 95%CI)	na	0.24 (− 0.20;0.67)	na	− 0.69^c^ (− 1.00;-0.39)	na	−0.81 (− 1.87; 0.26)
Bodyweight (kg)	na	0.17 (−1.44;1.77)	na	− 3.12^c^ (− 4.26;-1.99)	na	− 2.16 (− 5.49;1.17)
Work characteristics:						
Work ability (0–10) (mean, 95%CI)	− 1.89 (− 0.43;0.05)	−0.18 (− 0.45;0.09)	−0.02 (− 0.20;0.16)	−0.11 (− 0.35;0.13)	0.08 (− 0.19;0.36)	−0.01 (− 0.47;0.46)
Sickness absence (%)						
≥10 days	2.4	10.9^c^	−1.4	− 1.5	−1.6 (− 7.0;5.2)	−7.2 (− 15.5;1.2)
Productivity loss (%, 95CI)	1.84^c^ (1.15;2.53)	2.31^c^ (1.56;3.07)	1.46^c^ (1.00;1.93)	1.47^c^ (0.94;2.00)	−0.17 (− 1.07;0.73)	−0.44 (− 1.80;0.92)
Health risk behaviour:						
Lack of physical activity (%)	−58.6^c^	−53.6^c^	−49.2^c^	−50.3^c^	−6.5 (− 14.6;5.2)	−5.6 (− 14.2;5.0)
Smoking (%)	−4.6	−3.2	−2.3	0	10.5 (2.4;15.5)	8.6 (−0.1;15.7)
Excessive alcohol use (%)	−5.2	−11.1^c^	−5.1^c^	−9.4^c^	2.0 (− 2.1;6.9)	0.0 (− 2.1;6.9)

*na* ‘not applicable’, *ref* reference, *SD* standard deviation

^a^Unadjusted

^b^Difference calculated with a mixed effects model and adjusted for age, gender, education, cluster and, in case of continuous outcome measures, also for baseline values

^c^*P* < 0.05

The extensive group attended 4 MI-sessions on average (SD 2.41) with a total mean duration of 104 min (SD 64.8). The analysis on delivery of the intervention showed that, on average, the level of received MI was 3.5 for empathy (SD 0.54) and 83.7% was delivered at sufficient MI-adherence (SD 10.25).

## Discussion

The results of this study show no effects on self-rated health, BMW, and body weight. The effects and sustainability of weight loss by adding MI-coaching to a web-based HRA among employees at increased CVD-risk in the military workforce, the police organisation and an academic hospital were promising, albeit not statistically significant. Both in the extensive and limited intervention group the proportion of subjects who engaged sufficiently in physical activity increased sharply, productivity loss increased, and excessive alcohol use declined.

The additional reduction of 0.81 kg/m^2^ in BMI (3.1 kg or 3.2% body weight loss) by the extensive intervention group compared to the limited group is high compared to other CVD-risk reducing interventions. Beishuizen et al. [36] showed in a meta-analysis of 47 studies a mean reduction in body weight of 1.3 kg in web-based interventions in individuals older than 50 years and at increased risk for CVD. While MI was associated with a significant reduction in body weight of 1.5 kg in overweight and obese individuals [37], no effect of MI on BMI was found in a pooled analysis of 3 studies that included individuals at increased CVD risk [12]. Nevertheless, the effects on body weight found in our trial are even more pronounced than the 1.8 kg difference between intervention groups in Groeneveld’s study [38], which was most similar to ours in terms of target population and intervention. Our study differed in that a web-based HRA with personalized and tailored feedback was provided and used as starting point for counselling instead of just a cardioscreening. The reduction in BMI could be due to targeting multiple health behaviours, which was found to be more effective than focussing on just one component [39]. Although it has been shown that effects are more pronounced in studies with shorter follow-up time [36], both Groeneveld et al. [38] and our study also showed reductions in BMI after 12 months.

In contrast to the effects on BMI and body weight, the effects on productivity loss, smoking and physical activity in our study are harder to interpret. In contrast to previous studies reporting that risky behaviour was associated with increased productivity loss at work [40], our study showed an increased productivity loss at follow-up, while health behaviour improved. This increase of productivity loss may be related to major national reorganizations in both the police and the military during the study period, with consequent work-related stress leading to productivity loss [41]. Our results on smoking cessation following a workplace intervention are in line with other studies [42]. We observed a smaller effect in the extensive compared to the limited group, which is in contrast to abundant evidence by others [43, 44]. Since previous research has shown that smoking is better targeted as the primary or only outcome instead of being integrated in a programme targeting multiple risk factors [42], this most likely explains our results. The sharp increase in the proportion of subjects meeting the Dutch guideline for physical activity cannot be attributed to the motivational interviewing, given that physical activity improved in both randomised groups. Nevertheless, the HRA result may have acted as a warning signal that subjects needed to improve rapidly in particular in the military and police where a good physical condition is a prerequisite for the job. This may also explain the strong decrease in excessive alcohol use in both intervention groups.

There are several possible reasons why the intervention showed a statistically significant effect on BMI compared to baseline in the extensive group but no statistically significant differences on other outcomes. Several issues may have reduced the beneficial effects of the extensive intervention, such as methodological issues, insufficient delivery of the intervention, or ineffectiveness for certain outcomes. The methodological limitation is linked to our cluster design with large cluster-size differences (ranging 1–124), which may have caused under-powering of the study. A linked issue is that a cluster RCT is sensitive to allocation bias, as was indeed present as illustrated by the disbalance in age, gender, and education at baseline between the extensive and limited intervention groups. Adjustment for these factors led to the lack of precision in the estimated effect of the extensive intervention group compared to the limited intervention group. Concerning the delivery of MI, both quantity and quality as provided by OPs need to be considered. Since the prescribed dose of 7 MI-sessions was not met by 75% of the individuals, whereas BMI decreased statistically significantly, this may suggest that the optimum MI-dose is lower than 7 or, alternatively, that this is determined by personal needs rather than one-size-fits-all. This is in line with the inconclusiveness in previous publications on the optimal dose restricted to individuals at risk or diagnosed with CVD [11], creating the need for future research focusing on what is the optimal dosage for whom. The quality of MI in this study appeared fairly low according to the MITI thresholds [35], with an insufficient level of MI-adherence and empathy at beginner’s level. Since the awareness of the quality of MI as a factor in effectiveness of MI has grown [45], a more detailed exploration of MI-fidelity is needed [34, 45].

A potential limitation is that the intervention has failed to target individuals who needed it most, based on low work ability and high productivity loss at work. However, the average response rate of 77.8% in this study was high compared to 33% in other studies [46]. A second limitation is that this study lacked a third arm including a non-intervention-group. Although this means that changes in the intervention groups are not necessarily a result of the HRA, there is sufficient evidence that a purely web-based HRA impacts health and work at least in the short-term [6–9]. A third limitation is that the PerfectFit intervention might increase the participants’ motivation to change, but not sufficiently to change their behaviour, resulting in an underestimation of the effect. This idea is strengthened by increased adherence to follow-up in the extensive group. A linked issue is that the three organisations may differ in organizational support for healthy behavior, but in the current study it was not possible to evaluate the influence of the organisation on participants’ behavior. Individual health behaviour change is mediated by a multitude of factors [47], including a more job-specific approach [48] and involving multiple levels of the workplace such as management and colleagues [49], could improve the individual’s work and health outcomes.

Strengths of our study are the performance in a real-life setting, the assessment of the additional effect of supplementing a web-based HRA with tailored advice and face-to-face coaching on both clinical and societal outcomes, and the assessment of sustainability by prolonged follow-up. Many interventions are effective in controlled research settings, but to achieve scaling-up such interventions they must be embedded within multiple sectors [50]. Since our study was performed in a real-life setting, in a multi-center approach in different sectors, and by the OPs who are working in these organisations, our findings may be generalisable to other organisations and applied in future implementation.

## Conclusions

There were no effects on self-rated health, body weight, and BMI. However, within the group with web-based tailored Health Risk Assessment including personalized advice body weight reduced significantly. Adding personalized coaching to a web-based HRA in a ‘blended care’-approach is promising in the reduction of BMI and body weight in employees at increased CVD risk. Future research may be aimed towards a) personalised prediction modelling to determine who will benefit optimally from a web-based HRA and who will need additional coaching, and b) the influence of a supportive work environment.

## Additional files

Additional file 1: **Table S1.** CONSORT 2010 checklist for reporting a cluster randomised trial & Extension of CONSORT for abstracts to reports of cluster randomised trials. **Table S2.** Extension of CONSORT for abstracts to reports of cluster randomised trials. (DOCX 33 kb)

Additional file 2: **Table S3.** Characteristics of non-responders at 6 or 12 months follow-up (*n* = 109). (DOCX 22 kb)

## Abbreviations

BMI: Body Mass Index
CVD: Cardiovascular disease
HRA: Web-based health risk assessment
MI: Motivational interviewing
OP: Occupation Health Physician
PA: Physical Activity
