# Supplementary material for: Effectiveness of the blended-care lifestyle intervention ‘PerfectFit’: a cluster randomised trial in employees at risk for cardiovascular diseases
Source: BMC Public Health. 2018 Jun 19;18:766. doi: 10.1186/s12889-018-5633-0 (PMC6009059; doi:10.1186/s12889-018-5633-0)
Supplement: Supplementary file 2 — Table S3. Characteristics of non-responders at 6 or 12 months follow-up (n = 109). (DOCX 22 kb) [file 12889_2018_5633_MOESM2_ESM.docx]

Additional file 2: Table S2. Characteristics of non-responders at 6 or 12 months follow-up (n=109).

| Baseline characteristics: | Responded at follow-up  n= 382 (77.8%) | Did not respond at follow-up  n=109 (22.2%) |
| --- | --- | --- |
| Intervention-group | | |
| Limited | 155 (40.6)^e^ | 62 (56.9)^e^ |
| Extensive | 227 (59.4)^e^ | 47 (43.1)^e^ |
| Organisation | | |
| Military | 202 (52.9) | 60 (55.0) |
| Police | 137 (35.9) | 33 (30.3) |
| Hospital | 43 (11.3) | 16 (14.7) |
| Individual characteristics: | | |
| Age, years (mean, SD) | 50.84 (5.50) | 50.74 (6.33) |
| Gender (n,%): | | |
| Male | 311 (81.4) | 88 (80.7) |
| Female | 71 (18.6) | 21 (19.3) |
| Level of education (n,%): | | |
| Low | 51 (13.4) | 16 (14.7) |
| Medium | 208 (54.5) | 48 (44.0) |
| High | 108 (28.3) | 20 (18.3) |
| Health characteristics: | | |
| Self-rated general health: |  |  |
| Less than good | 70 (18.3) | 20 (18.3) |
| Body mass index (kg/m^b^)(mean, SD) | 27.2 (3.6) | 27.2 (3.0) |
| Work characteristics: | | |
| Working hours per week (mean, SD) | 37.4 (5.8) | 36.3 (5.5) |
| Work ability (mean, SD) | 8.0 (1.4)^e^ | 7.5 (1.9)^e^ |
| Sickness absence |  |  |
| 0 days | 187 (49.0) | 28 (25.7) |
| 1-9 days | 141 (36.9) | 37 (33.9) |
| ≥10 days | 36 (9.4) | 9 (8.3) |
| Productivity loss at work (%, SD) | 2.7 (3.3)^e^ | 4.2 (4.3)^e^ |
| Health risk behaviour^a,^ n (%): | | |
| Lack of physical activity | 261 (68.3) | 60 (55.0) |
| Unhealthy diet^b^ | 352 (92.1) | 73 (67.0) |
| Smoking | 62 (16.2) | 21 (19.3) |
| Excessive alcohol use^c^ | 51 (13.4) | 7 (6.4) |
| High stress level^d^ | 141 (53.9) | 24 (22.0) |
| Intervention-group only: Characteristics of the intervention | | |
| MI-sessions (mean, SD) | 4.5 (2.2)^e^ | 1.4 (1.6)^e^ |
| ≥4 MI-sessions (n,%) | 150 (39.3)^e^ | 5 (4.6)^e^ |
| Duration (mins) (mean, SD) | 117.7 (60.2)^e^ | 41.1 (45.4)^e^ |
| Empathy (1-5)(mean, SD) | 3.5 (0.52) | 3.6 (0.62) |
| MI-adherence (%, SD) | 83.7 (9.7) | 83.9 (12.7) |

**^a^**Defined as no adherence to Dutch guidelines at baseline.

**^b^**Unhealthy diet is defined as eating less than 200 g vegetables per day , and eating less than 2 pieces of fruit per day

**^c^**Meeting the alcohol guideline, which is not drinking more than 1 (women) or 2(men) glasses of alcohol a day

**^d^**High stress level is defined as several periods or permanent stress at work or at home or severe financial stress or 2 or more life events (Ref. Lancet 2004 Rosengren).

BMI: body mass index

SD: standard deviation

^e^*P*<0.05
